# *In vitro* Characterization of the Regional Binding Distribution of Amyloid PET Tracer Florbetaben and the Glia Tracers Deprenyl and PK11195 in Autopsy Alzheimer’s Brain Tissue

**DOI:** 10.3233/JAD-201344

**Published:** 2021-04-20

**Authors:** Ruiqing Ni, Jennie Röjdner, Larysa Voytenko, Thomas Dyrks, Andrea Thiele, Amelia Marutle, Agneta Nordberg

**Affiliations:** a Division of Clinical Geriatrics, Center for Alzheimer Research, Department of Neurobiology, Care Sciences and Society, Karolinska Institutet, Stockholm, Sweden; b Bayer Pharma AG, Berlin, Germany; c Theme Aging, The Aging Brain Unit, Karolinska University Hospital, Stockholm, Sweden

**Keywords:** Alzheimer’s disease, amyloid-beta peptides, astrocytes, glial fibrillary acid protein, microglia, monoamine oxidase B, positron emission tomography

## Abstract

**Background:**

Emerging evidence indicates a central role of gliosis in Alzheimer’s disease (AD) pathophysiology. However, the regional distribution and interaction of astrogliosis and microgliosis in association with amyloid-β (Aβ) still remain uncertain.

**Objective:**

Here we studied the pathological profiles in autopsy AD brain by using specific imaging tracers.

**Methods:**

Autopsy brain tissues of AD (*n* = 15, age 70.4±8.5 years) and control cases (*n* = 12, age 76.6±10.9) were examined with homogenate binding assays, autoradiography for Aβ plaques (^3^H-florbetaben/^3^H-PIB), astrogliosis (^3^H-L-deprenyl), and microgliosis (^3^H-PK11195/^3^H-FEMPA), as well as immunoassays.

**Results:**

*In vitro* saturation analysis revealed high-affinity binding sites of ^3^H-florbetaben, ^3^H-L-deprenyl, and ^3^H-PK11195/^3^H-FEMPA in the frontal cortex of AD cases. *In vitro* ^3^H-florbetaben binding increased across cortical and subcortical regions of AD compared to control with the highest binding in the frontal and parietal cortices. The *in vitro* ^3^H-L-deprenyl binding showed highest binding in the hippocampus (dentate gyrus) followed by cortical and subcortical regions of AD while the GFAP expression was upregulated only in the hippocampus compared to control. The *in vitro* ^3^H-PK11195 binding was solely increased in the parietal cortex and the hippocampus of AD compared to control. The ^3^H-florbetaben binding positively correlated with the ^3^H-L-deprenyl binding in the hippocampus and parietal cortex of AD and controls. Similarly, a positive correlation was observed between ^3^H-florbetaben binding and GFAP expression in hippocampus of AD and control.

**Conclusion:**

The use of multi-imaging tracers revealed different regional pattern of changes in autopsy AD brain with respect to amyloid plaque pathology versus astrogliosis and microgliosis.

## INTRODUCTION

Alzheimer’s disease (AD) involves complex pathophysiology, featured by amyloid-β (Aβ) deposits, neurofibrillary tangles, gliosis, and neural loss. Emer-ging evidence implies that Aβ, astrogliosis, and mi-crogliosis play important roles at early phases of AD [1–5]. However, the regional distribution, consequence of gliosis in relation to Aβ deposits, whether beneficial or harmful, as well as accurate imaging biomarker for gliosis remains elusive.

Autopsy analysis and *in vivo* positron emission tomography (PET) with Aβ tracers in AD and non-demented control cases [6] have shown that different brain regions develop Aβ pathology following the hierarchy of neocortical, limbic, and subcortical areas [7–9]. Higher cortical Aβ loads (retentions) were observed in patients with AD and mild cognitive impairment (MCI) due to AD compared with control [6, 10] by PET using Aβ tracers such as ^11^C-PIB [11], ^18^F-flutemetamol [12], ^18^F-florbetapir [13], ^18^F-florbetaben [14–16], and ^11^C-AZD2184/^18^F-AZD4694 [17]. Robust correlations between PET and Aβ pathology at autopsy support the specific detection by Aβ imaging tracers [18–22]. ^18^F-florbetaben binding *in vivo* correlated strongly with neuritic plaques detected by Bielschowsky-silver staining and 6E10 immunostaining in AD brain [14], and showed no binding to alpha-synuclein or tau pathologies [23]. The *in vitro* regional distribution of ^3^H-florbetaben in autopsy AD brain remains to be characterized.

Accurate detection of neuroinflammation and gliosis *in vivo* has been challenging [4, 24]. One reason is that astrocytes and microglia are highly dynamic and heterogeneous in their subtypes, locations, and activation statuses [25]. ^11^C-deuterium-L-deprenyl (DED) binds to monoamine oxidase-B (MAO-B) overexpressed in reactive astrocytes and has been evaluated as a biomarker for astrogliosis [26–29]. ^11^C-DED binding increased in MCI with high ^11^C-PIB retention compared to AD and control subjects. Longitudinal study in patients with autosomal \nobreak dominant AD showed that ^11^C-DED binding elevated at initial disease stage and declined with increasing ^11^C-PIB binding during progression, suggesting astrogliosis as an early event [3, 30].

Microglia activation has been assessed by PET using translocator protein (TSPO) tracers [31, 32], such as first-generation ^11^C-PK11195 [33], second-generation ^11^C-DAA1106 [34], ^11^C-PBR28 [35], ^18^F-FEMPA [36], ^11^C-GE180 [37], and ^18^F-DPA-714 [38]. Results from TSPO imaging in MCI and AD have been inconclusive: increase or no change in TSPO retention comparing AD to control group has been reported [33, 39–41]. This could be due to several reasons, including 1) tracer specificity [42]; 2) *TSPO rs6971* genetic polymorphism [25, 43]; 3) heterogeneous and dynamic activation status of microglia [44]; and 4) various cellular expression of TSPO.

To understand the regional distribution of fibrillar Aβ deposits in relation to astro- and microgliosis, we determined the binding of ^3^H-florbetaben/^3^H-PIB, ^3^H-L-deprenyl, and ^3^H-PK11195/^3^H-FEMPA in autopsy brain tissues from AD and control cases by using homogenate binding assays, autoradiography, enzyme-linked immunosorbent assay (ELISA) for astrocyte marker glial fibrillary acidic protein (GFAP). This study demonstrates a clear regional correlation between amyloid plaque deposition and astrogliosis in AD.

## MATERIALS AND METHODS

### AD and control autopsy brains

Fifteen AD cases (mean age 70.4±8.5 years; mean postmortem delay 5.1±0.9 h), each with a clinical diagnosis confirmed by pathological examination (NINCDS-ADRDA criteria), and twelve control cases (mean age 76.6±10.9 years; mean postmortem delay 7.0±3.8 h) were included in this study (Table 1). Early-onset AD (EOAD) and late-onsetAD (LOAD) were classified based on the age of on-set of the clinical symptoms (before or after 65 years of age). Autopsy brain tissues from the frontal, parietal and temporal cortics, hippocampus, caudate nucleus, and cerebellum were obtained from the Netherlands Brain Bank (NBB), Netherlands. The disease relevant cortical and subcortical brain regions, as well as the reference brain region in amyloid PET imaging (cerebellum) were selected. All materials had been collected from donors or from whom a written informed consent for a brain autopsy and the use of the materials and clinical information for research purposes had been obtained by the NBB. Frozen brain tissues from the left hemisphere (all cases) were homogenized in ice-cold 5×0.32 M sucrose containing 10μl/ml protease inhibitor; brain tissues from 4 AD and 4 control cases were cryostat sectioned at 10μm and stored at –80°C. Protein concentration was determined using DC protein assay (Bio-Rad Laboratories AB, Sweden). The apolipoprotein E (*APOE*) genotype was determined using an INNO-LIPA ApoE-kit (Innogenetics, Belgium) with genomic DNA extracted from the thalamic tissue of AD and control cases (QIAamp DNA mini kit, Qiagen, Germany).

**Table 1:** Demographics

Group	No. of brains	Age (y)	Sex (F/M)	Disease duration (y)	PM delay (h)	*APOE* *ɛ*4 (0/1/2)	Braak stage (0–6)
AD	15	70.4±8.5	9/6	6.7±2.7	5.1±0.9	4/5//6	4/5/6
EOAD	8	63.8±5.4	5/3	7.4±2.3	4.8±0.9	3/3/2	4/5/6
LOAD	7	78.0±4.4	4/3	5.7±3.0	5.4±0.9	1/2/4	4/5/6
Control	12	76.6±10.9	6/6		7.0±3.8	12/0/0	1/2

EOAD, early onset Alzheimer’s disease; LOAD, late onset Alzheimer’s disease; PM, postmortem.

The study was conducted according to the principles of the Declaration of Helsinki and subsequent revisions. All experiments on autopsied human brain tissue were carried out in accordance with ethical permission obtained from the regional human ethics committee in Stockholm (permission number 2011/962/31-1), the medical ethics committee of the VU Medical Center for the Netherlands Brain Bank tissue (permission no. 1998-06/5).

### Materials

^3^H-florbetaben ([N-*methyl*-^3^H]4-[(E)-2-(4-{2-[2-(2-fluoroethoxy)ethoxy]ethoxy}phenyl) ethenyl]-aniline), specific activity(SA) 63.0 Ci/mmol; ^3^H-FEMPA ([N-*methyl*-^3^H][2-(2-Fluoroethoxy)-5-met-hoxybenzyl]-N-{2-[(4-methoxyphenyl)oxy]pyridin-e-3-yl}-acetamide), SA 38.7 Ci/mmol; and unlabeled florbetaben were custom synthesized by Bayer AG, Germany. ^3^H-L-deprenyl (N-Methyl-^3^H Hydro-chloride), SA 80.0 Ci/mmol, was purchased from American radiolabeled chemicals, USA. ^3^H-PK11195 (1-(2-Chlorophenyl)-N-methyl-N-(1-methylpro-pyl)-3-isoquinolinecarbox-amide), SA 81.7 Ci/mmol, was purchased from Perkin Elmer, USA. ^3^H-PIB [N-*methyl*-^3^H]2-(4’-Methylaminophenyl)-6-hydroxybenzo-thiazole), SA 85.0 Ci/mmol, was custom synthesized by GE Healthcare, UK. Unlabelled L-deprenyl, unlabelled PK11195 and 2-(4’-Methylaminophenyl)benzothiazole (BTA-1), bovine serum albumin (BSA) were purchased from Sigma-Aldrich, USA.

### Characterization of ^3^H-florbetaben, ^3^H-L-deprenyl, ^3^H-FEMPA, and ^3^H-PK11195 binding properties in AD and control brains

Saturation binding assays with ^3^H-florbetaben (SA 63.0 Ci/mmol) were performed in the frontal cortex homogenates of AD (*n* = 3) and control cases (*n* = 3) by incubation in 0.1 M PBS + 0.1 % BSA buffer (pH 7.4) containing 0.01–40 nM ^3^H-florbetaben for 3 h in tubes at room temperature (RT). Incubations were terminated by filtering samples through Whatman GF/C glass filters pre-soaked with 0.3% polyetylenamine solution. The filters were then washed 3×ice-cold 0.1 M PBS buffer (pH 7.4), transferred to scintillation vials and the radioactivity counted in a LS-6500 liquid scintillation counter (Beckman Coulter AB, Sweden) [45]. All assays were run in triplicates. The ^3^H-florbetaben specific binding was calculated as the difference between total (Non-specific (NSP) binding in the presence of 0.75μM unlabelled florbetaben) and expressed as pmol/g tissue.

Saturation binding assays with ^3^H-L-deprenyl (SA 80.0 Ci/mmol) were performed in the frontal cortex homogenate of AD cases (*n* = 7) by incubation in ice-cold 0.1 M Na^+^-K^+^ buffer (pH 7.4) containing 0.01–25 nM ^3^H-L-deprenyl for 2 h at 37°C (NSP binding in the presence of 1μM unlabelled L-deprenyl). The termination of incubation and counting is the same as aforementioned, except washing 3×ice-cold 0.1 M Na^+^-K^+^ buffer (pH 7.4). Saturation binding assays with ^3^H-PK11195 (SA 81.7 Ci/mmol) and ^3^H-FEMPA (SA 38.7 Ci/mmol) were performed in the frontal cortex homogenates of AD cases (*n* = 6) by incubation in 0.1 M Tris-HCl buffer (pH 7.4) containing 0.1–30 nM ^3^H-FEMPA or 0.5–120 nM ^3^H-PK11195 for 2 h at RT (NSP binding in the presence 10μM unlabeled PK11195). The termination of incubation is the same as aforementioned, except washing 3×ice-cold 0.1 M Tris-HCl buffer (pH 7.4). The specific binding of ^3^H-L-deprenyl, ^3^H-FEMPA and ^3^H-PK11195 were calculated as the difference between total and NSP binding and expressed as pmol/g protein.

Measurements of regional ^3^H-florbetaben (5 nM), ^3^H-L-deprenyl (6 nM), ^3^H-PK11195 (2.5 nM), and ^3^H-FEMPA (2.5 nM) binding were performed in the frontal, parietal, temporal cortex, hippocampus, caudate nucleus, and cerebellum of 12 AD and 12 control cases. The incubation time, incubation buffer, and washing buffers were same as in aforementioned saturation assay respectively. Measurement of ^3^H-PIB (1 nM, SA 85.0 Ci/mmol) binding was performed in the frontal and parietal cortex of the same 12 AD and 12 control cases by incubating in 0.1 M PBS + 0.1% BSA buffer (pH 7.4) for 2 h at RT. NSP binding was determined in the presence of 1μM BTA-1. The termination and counting procedure for ^3^H-PIB is the same as that for ^3^H-florbetaben assay. The ^3^H-PIB specific binding was calculated as the difference between total and NSP binding and expressed as pmol/g tissue.

### ELISA measurement of GFAP level

The level of GFAP expression was determined by using ELISA as described previously [46] in the frontal, parietal, temporal cortex, hippocampus, caudate nucleus of 12 AD and 12 control cases. Brain tissue homogenates were sequentially incubated with monoclonal anti-GFAP antibody (Merck KGaA, Germany), alkaline phosphatase-conjugated anti-mouse IgG (Vector, USA), AP and substrate (p-nitro-phenyl-phosphate; Bio-Rad) in a rabbit anti-GFAP antibody pre-coated (Dako, Denmark) microtiter plate. Optical density was assessed in a microtiter plate reader (Tecan, Switzerland) at 405 nm. The level of GFAP was analyzed using Soft Max Pro Plus software (Molecular Devices, USA).

### Autoradiography using ^3^H-florbetaben, ^3^H-L-deprenyl and ^3^H-FEMPA in AD brain tissue slices

Adjacent slices (10μm) from the frontal cortex (*n* = 4 each) and hippocampus (*n* = 3 each) of AD and control were dried in RT for 20 min and incubated with 2.5 nM ^3^H-florbetaben in 0.1 M PBS + 0.5 % BSA buffer (pH 7.4) buffer, 10 nM ^3^H-L-deprenyl, and 2.5 nM ^3^H-FEMPA in 0.1 M Tris-HCl buffer (pH 7.4) for 1 h with NSP binding determined in the presence of unlabeled 0.75μM florbetaben, 1μM L-deprenyl, and 10μM PK11195, respectively. After incubation, the brain slices were washed in corresponding ice-cold buffer 2×5 min and once in ice-cold dd water. The sections were dried in the hood and were exposed to Fuji BAS-TR2040 phosphor imaging plates (Science Imaging Scandinavia AB, Sweden) together with tritium microscale \nobreak standards (American Radiolabeled Chemicals); 4 days for ^3^H-florbetaben, 20 days for ^3^H-FEMPA, and 10 days for ^3^H-L-deprenyl. The plates were developed with a Fujifilm BAS-5000 phosphorimager (Fuji, Japan). Binding density was measured using Multigauge software V3.0 (Fuji, Japan).

### Statistical analyses

Data was analyzed using GraphPad Prism software 7.0 (GraphPad Software Inc, USA). K_d_ and B_max_ values from saturation binding curves were determined using a one-site non-linear fitting model. Non-parametric *t*-test (Mann-Whitney) were used for comparisons between AD and control cases. Two-way ANOVA was used to compare early onset AD, late onset AD and control group. Correlation analyses were performed using Pearson’s r correlation analysis. Significance level was set at ^*^p < 0.05, ^**^p < 0.01, ^***^p < 0.001.

## RESULTS

### Demographic information

Fifteen AD (9 female/6 male) and twelve control cases (6 female/6 male) were included in this study (Table 1). Among the AD cases, 8 were EOAD and the other 7 were LOAD cases. Among the control cases, 2 were younger than 65 years old, and 10 were older than 65 years old. Age and gender did not significantly differ between AD and control groups analyzed by using *Chi*-squared test. *APOE* *ɛ*4 genotyping results showed that AD group consisting of seven *ɛ*4/4 carriers, five *ɛ* 3/4 carriers, and four *ɛ*4 non-carriers. All control cases are *APOE* *ɛ*4 non-carriers.

### Binding characterization

Saturation binding studies with ^3^H-florbetaben at 0.01–40 nM revealed a high-affinity binding site of K_d_ = 11.8±1.4 nM in the frontal cortex of AD by non-linear fitting (Table 2). Saturation binding studies with ^3^H-L-deprenyl at 0.01–25 nM revealed a high-affinity binding site of K_d_ = 10.2±0.8 nM in the frontal cortex of AD cases (*n* = 7) (Table 2). The non-specific binding was approx. 5% for ^3^H-L-deprenyl. Saturation binding studies with ^3^H-PK11195 at 0.5–40 nM and ^3^H-FEMPA at 0.1–30 nM revealed similar high-affinity binding site of 1.8±0.6 nM and 3.3±0.1 nM respectively in the frontal cortex of AD cases (*n* = 6) by non-linear fitting (Table 2, Supplementary Figure 1). The non-specific binding was less than 10% for both ^3^H-PK11195 and ^3^H-FEMPA.

**Fig. 1:**
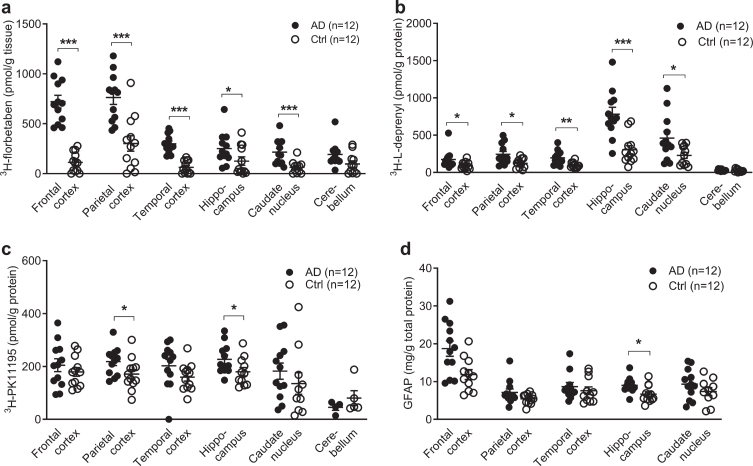
Regional ^3^H-florbetaben, ^3^H-L-deprenyl, ^3^H-PK11195 binding and level of GFAP expression in AD and control brain. a) ^3^H-florbetaben (5 nM) binding in the brain homogenate from AD and control cases. b) ^3^H-L-deprenyl (6 nM) binding in brain tissue homogenates from AD and control cases. c) ^3^H-PK11195 (2.5 nM) binding in brain tissue homogenates from AD and control cases. d) Level of GFAP expression in the same AD and control cases; Significant differences between AD and control groups are indicated by ^*^p < 0.05, ^**^p < 0.01, ^***^p < 0.001.

**Table 2:** Binding properties for ^3^H-florbetaben, ^3^H-L-deprenyl, ^3^H-FEMPA, and ^3^H-PK11195 in the frontal cortex of AD cases

Tracer	K_d_ (nM)	B_max_ (pmol/g tissue or protein)
^3^H-florbetaben (*n* = 3)	11.8±1.4	2146±964
^3^H-L-deprenyl (*n* = 7)	10.2±0.8	322±47
^3^H-FEMPA (*n* = 6)	3.3±0.1	251±147
^3^H-PK11195 (*n* = 6)	1.8±0.6	355±139

K_d_, dissociation constant; B_max_, maxium number of binding sites. Results are expressed as mean±standard deviation.

### Different regional ^3^H-florbetaben, ^3^H-L-deprenyl, ^3^H-PK11195, and GFAP distribution in AD and control brain

^3^H-florbetaben (5 nM) showed significant increa-ses in the frontal cortex (*p* < 0.001), followed by the parietal cortex (*p* < 0.001), temporal cortex (*p* < 0.001), caudate nucleus (*p* < 0.001), and hippo-campus (*p* = 0.0387) of AD (*n* = 12) compared to control (*n* = 12) (Fig. 1a). ^3^H-L-deprenyl (6 nM) showed significant increases in the hippocampus (*p* = 0.0002) followed by the temporal cortex (*p* = 0.0014), the frontal and parietal cortex (*p* = 0.0284, *p* = 0.0121), and caudate nucleus (*p* = 0.0374) of AD (*n* = 11-12) compared to control cases (*n* = 12) (Fig. 1b). 2.5 nM ^3^H-PK11195 showed significantly increases in the parietal cortex (*p* = 0.0332) and hippocampus (*p* = 0.0317) of AD (*n* = 12) compared with control (*n* = 12) (Fig. 1c). In comparison, another TSPO tracer ^3^H-FEMPA (2.5 nM) did not show difference in any brain regions between AD and control (Supplementary Figure 1). The levels of GFAP expression were determined by ELISA in five brain regions of the same AD (*n* = 12) and control (*n* = 12) cases included in binding assays. The levels of GFAP expression significantly increased in the hippocampus (*p* = 0.0280) of AD compared to control (Fig. 1d), and not in the other regions.

The difference between EOAD, LOAD, and control group was analyzed using two-way ANOVA comparison (Table 3). High ^3^H-florbetaben binding was observed in in all three cortical regions in EOAD and LOAD compared to control. The ^3^H-L-deprenyl binding was higher in the hippocampus while lower in the caudate nucleus of LOAD compared to EOAD. No difference was observed in the regional ^3^H-florbetaben, or ^3^H-PK11195 binding between EOAD and LOAD group.

**Table 3:** Comparison of ^3^H-florbetaben, ^3^H-L-deprenyl, ^3^H-FEMPA, and ^3^H-PK11195 binding between EOAD, LOAD and control

Tracer	Region	Group			p		
		EOAD (*n* = 7)	LOAD (*n* = 5)	Ctrl (*n* = 12)	EOAD versus Ctrl	LOAD versus Ctrl	EOAD versus LOAD
^3^H-L-deprenyl	PC	187±66	322±175	138±55	ns	0.0183	ns
	HIP	662±214	946±387	301±191	< 0.0001	< 0.0001	0.0004
	CN	546±381	342±153	245±125	< 0.0001	ns	0.0168
^3^H-florbetaben	FC	737±210	698±256	113±100	< 0.0001	< 0.0001	Ns
	PC	804±200	705±302	302±269	< 0.0001	< 0.0001	Ns
	TC	315±95	277±97	64±64	0.0001	0.0047	Ns
	HIP	274±171	210±118	125±145	0.0366	ns	Ns

CN, caudate nucleus; Ctrl, control; EOAD, early onset Alzheimer’s disease; HIP, hippocampus; LOAD, late onset Alzheimer’s disease; ns, not significant; PC, parietal cortex; TC, temporal cortex. Values represent Mean±standard deviation.

### Autoradiography showed high ^3^H-florbetaben in the cortex, and ^3^H-L-deprenyl signal in the dental gyrus

**Fig. 2:**
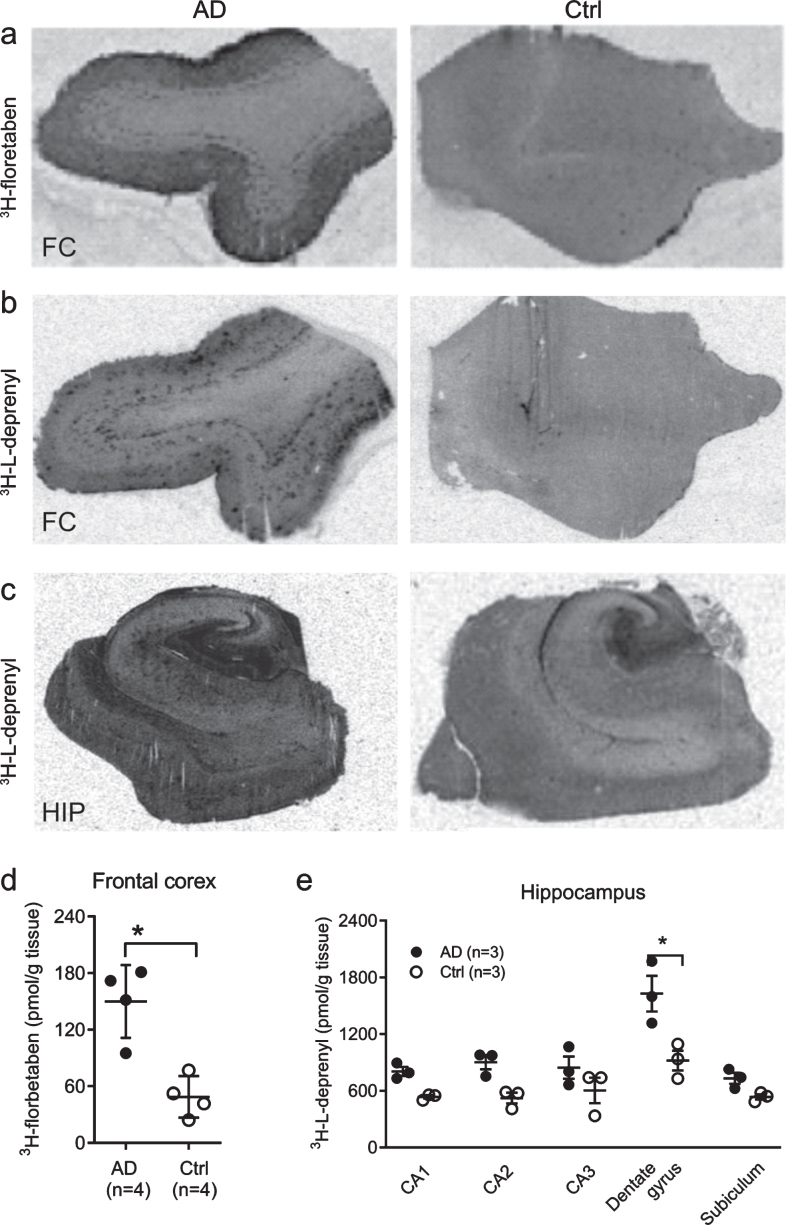
Autoradiography using ^3^H-florbetaben and ^3^H-L-deprenyl in AD and control brain. Autoradiography and quantification in AD (*n* = 3) and control cases (*n* = 3), using (a, d) ^3^H-florbetaben (5 nM) in the frontal cortex slices; (b-c, e) using ^3^H-L-deprenyl (10 nM) in the frontal cortex and the hippocampus slices; Significant differences between AD and control groups are indicated by ^*^p < 0.05.

Autoradiography using ^3^H-florbetaben (2.5 nM) showed increased signal in the grey matter in the frontal cortex slices from AD compared to control cases (*p* < 0.05) (Fig. 2a, d). To further analyze the sub-regional distribution of ^3^H-L-deprenyl binding, autoradiography using ^3^H-L-deprenyl (10 nM) was performed in the frontal cortex and hippocampus slices from three AD and three control cases (Fig. 2b, c). ^3^H-L-deprenyl binding was higher in the dentate gyrus of AD compared to control cases (*p* = 0.0306, Fig. 2e). A laminar pattern of ^3^H-L-deprenyl binding was observed in the frontal cortex (Fig. 2b) which was not observed for ^3^H-florbetaben (Fig. 2a).

### Correlation analysis

**Fig. 3:**
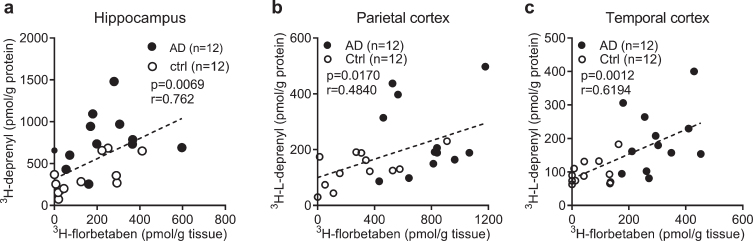
Correlation between regional ^3^H-florbetaben, ^3^H-L-deprenyl binding and level of GFAP expression in AD and control brain. a-c) Correlation between ^3^H-L-deprenyl (6 nM) and ^3^H-florbetaben (5 nM) binding in the hippocampus, parietal and temporal cortex of AD and control cases.

To investigate the relation between the levels of different readouts in AD and control, Pearson’s r correlation analysis was performed. The ^3^H-florbetaben binding positively correlated with ^3^H-L-deprenyl binding in the hippocampus, parietal and temporal cortex of AD and control cases (Fig. 3a-c). No significant correlation was observed between ^3^H-florbetaben and ^3^H-PK11195 binding in the hippocampus (data not shown). ^3^H-PIB (1 nM) binding measured in the frontal cortex and parietal cortex showed significant increases in AD (*n* = 12) compared to control cases (*n* = 12, *p* < 0.001); and correlating with ^3^H-florbetaben binding respectively (Supplementary Figure 2a, b).

**Fig. 4:**
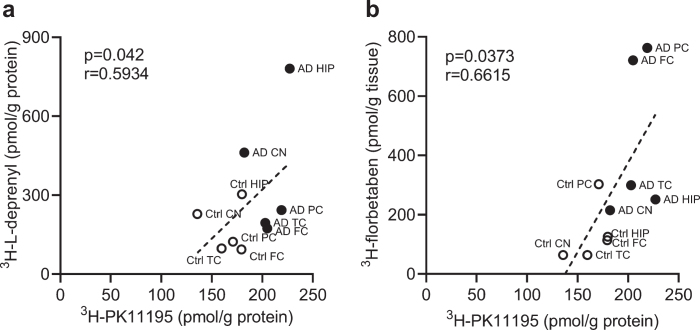
Correlation between regional ^3^H-PK11195, ^3^H-florbetaben, and ^3^H-L-deprenyl binding in AD and control brain. a) Correlation between ^3^H-PK11195 (2.5 nM) and ^3^H-L-deprenyl (6 nM) binding across the six brain regions: frontal cortex (FC), parietal cortex (PC), temporal cortex (TC), hippocampus (HC), caudate Nucleus (CN), and cerebellum (CN). b) Correlation between ^3^H-PK11195 (2.5 nM) and ^3^H-florbetaben (5 nM) binding across the five brain regions: FC, PC, TC, HC, and CN.

We analyzed the correlation between different readouts across the whole brain. Correlations were observed between ^3^H-PK11195 and ^3^H-L-deprenyl binding (*p* = 0.042, r = 0.5934) in the six brain regions (frontal, parietal, temporal cortex, hippocampus, caudate nucleus and cerebellum) as well as between ^3^H-PK11195 and ^3^H-florbetaben binding in AD and control groups across five brain regions (frontal, parietal, temporal cortex, hippocampus, and caudate nucleus) (Fig. 4a, b).

## DISCUSSION

Cellular events such as abnormal Aβ and tau ac-cumulation, dystrophic neurites, reactive astrocytes, and microglia contribute to the pathogenesis of AD. Different pathological subtypes of AD, with/without abnormal Aβ accumulation have been documented [2, 47, 48]. Regional characterization of different molecular pathology profiles in autopsy AD brain thus provides insights into the disease mechanism [49] as well as validation for imaging biomarkers. Here we demonstrated distinct region-spe-cific distributions of Aβ deposits (^3^H-florbetaben), astrogliosis (^3^H-L-deprenyl, GFAP) and activated microglia (^3^H-PK11195) in AD brain.

We showed that regional ^3^H-florbetaben binding corresponded to the known high cortical and low hippocampal Aβ deposits pattern in AD brain. Good *in vivo* ^18^F-florbetaben PET-Aβ histopathology correspondence, specific detection of cerebral Aβ deposits in AD [14, 51–53] has been reported from recent phase III studies. Our results of strong correlation of ^3^H-florbetaben and ^3^H-PIB binding is in line with reported comparable binding properties of the tracers [54, 55], and *in vivo* ^11^C-PIB and ^18^F-florbetaben PET head-to-head comparison results [56, 57]. The low hippocampal ^3^H-florbetaben binding in this study has been observed by *in vivo* PET using ^18^F-florbetaben [14] and ^11^C-PIB [58]. The amyloid tracer binding might differ from the pathological observations on the autopsy brain tissues that Aβ deposits accumulate in hippocampus at early phase II Thal stage [9]. One possible explanation is that the hippocampal Aβ deposits consist of more diffuse and oliogmeric Aβ with lower amount of β-sheet structures. Thus the β-sheet binding amyloid PET tracers show mainly low-affinity binding site as compared to the major high-affinity binding sites in the cortical AD brain tissues [9, 54].

Astrocytes play an important role in the brain physiology, as well as in learning and memory formation [59]. Reactive astrogliosis are dynamic and heterogeneous in their location, subtypes, hypertrophy/proliferation, and upregulation of different markers such as GFAP, vimentin, nestin, MAO-B, and gamma-amino butyric acid [60, 61]. Reactive astrocytes have been shown play an important role early in the development of AD [62, 63]. Elevated ^3^H-L-deprenyl (MAO-B) measures of astrogliosis was observed in the hippocampus especially in the dentate gyrus, followed by the cortical regions of AD, consistent with autopsy [50, 64, 65] and *in vivo* ^11^C-DED PET evidence [3]. The similar regional distribution pattern for amyloid plaques (^3^H-florbetaben) and astrogliosis (^3^H-L-deprenyl) may suggest a close relationship in AD pathology. However, a regional difference was observed with high astrogliosis measured in the hippocampus (dentate gyrus), a region with low amyloid plaque load. ^3^H-L-deprenyl showed a laminar binding pattern in the frontal cortex of AD which was absent for the amyloid plaque tracer ^3^H-florbetaben binding. This observation is consistent with earlier studies using ^3^H-L-deprenyl/^3^H-PIB [50] in the autopsy AD brain: ^3^H-L-deprenyl binding distributes in all layer in hippocampus and superficial layer in frontal cortex of AD brain, while ^3^H-PIB binding spreads in the all layers in the frontal cortex.

Regional GFAP expression seems to differ from ^3^H-L-deprenyl binding in AD brains. In comparison to ^3^H-L-deprenyl binding, the GFAP expression is most elevated in the frontal cortex of AD and a positive correlation with ^3^H-L-deprenyl was only observed in the hippocampus. Different distribution patterns of GFAP and ^3^H-L-deprenyl were also observed in brain of Tg2576 mouse model of AD [66]. These two methods might detect different status/types of astrogliosis, as 1) GFAP is expressed non-uniformly on subtypes of astrocytes [67]; 2) change in glia phenotype profiles but not number of GFAP + astrocytes in AD brain; 3) MAO-B expressed in astrocytes as well as in dopaminergic neurons mainly in subcortical regions.

We found similar high affinities of ^3^H-PK11195 and ^3^H-FEMPA in the cortex of AD, in line with reported high-affinity binding sites of ^3^H-PK11195 [43] and ^18^F-FEMPA [36]. The presence of subjects with mixed binding sites of ^3^H-FEMPA may lead to the observed larger variation in binding distribution compared to ^3^H-PK11195. ^3^H-PK11195 shows highest binding in the temporal cortex and hippocampus that is markedly affected by tau aggregates as shown by histopathology [68] and in PET investigations [69, 70]. ^11^C-PK11195 is the first PET tracer for TSPO imaging. Despite its limitations including a short half-life, relatively low brain uptake, suboptimal metabolic profile, high non-specific binding, ^11^C-PK11195 is still currently the most used TSPO tracer in AD research. The level of ^11^C-PK11195 and amyloid load measured by ^11^C-PIB in AD patients were positively correlated within the frontal, parietal and temporal cortices [71, 72]. Recent study using autopsy brain tissues suggest an overlap in the levels of TSPO protein and mRNA between AD and healthy-control, and a limited influence by *TSPO rs6971* polymorphism [43]. Novel TSPO tracers with improved binding specificity, SNR and higher brain uptake was pursued, such as ^18^F-GE-180 [73], ^11^C-PBR28 [74], ^11^C-DAA1106 [34], ^11^C-vinpocetine [75], ^18^F-DPA-714 [76], ^18^F-FEPPA [77], and ^11^C-AC5216 [78]. In addition, new tracers for imaging neuroinflammation beyond TSPO are under development for microgliosis [79, 80] and astrogliosis [4] (including novel MAO-B tracer [81]). Elucidating the cellular origin of the radiotracer binding such as TSPO binding [82] and whether they are selective for pro-inflammatory astrocytes and microglia will be critical [83].

We observe a similar higher regional ^3^H-florbetaben binding in EOAD and LOAD cases, compared to control cases. This is in line with previous observation by *in vivo* ^11^C-PIB imaging in EOAD and LOAD patients showing comparable amyloid load [84]. Interestingly the ^3^H-L-deprenyl binding was higher in the hippocampus of LOAD compared to EOAD, and lower in the caudate nucleus of LOAD compared to EOAD. For cerebral glucose metabolism (^18^F-FDG PET), a more pronounced hypometabolism has been reported in EOAD compared to LOAD [84]. Similarly, a higher *in vivo* binding of tau PET tracers as well as *in vitro* tau tracer binding have been observed in EOAD compared to LOAD patients [85]. Both FDG and tau tracer binding could be explained by a more pronounced AD pathology in EOAD compared to LOAD. It can be speculated that the finding in this study of higher astrogliosis (higher ^3^H-L-deprenyl binding) in the hippocampus of LOAD compared to EOAD might be due to more hippocampus sparing cases [86] in the EOAD group which might explain the lower astrogliosis in EOAD compared to LOAD. An increased ^11^C-DED binding has also been demonstrated in brain by PET in aging [87].

Positive correlations between ^3^H-florbetaben-GFAP and ^3^H-florbetaben-^3^H-L-deprenyl were observed in the hippocampus, parietal cortex, and temporal cortex in the current study. This corresponds to the reported ^3^H-PIB-GFAP correlation in sporadic AD autopsy brain [19]. However, negative Aβ_40_-GFAP correlation in both sporadic and familial AD have also been reported [49]. In addition, ^3^H-PK11195 binding positively correlates with ^3^H-florbetaben in five brain regions and ^3^H-L-deprenyl binding in all six brain regions. This corresponds to the reported positive ^11^C-PIB-^11^C-PK11195 correlation in AD cases [88]. The regional link between ^3^H-florbetaben-^3^H-L-deprenyl appears stronger than ^3^H-florbetaben-^3^H-PK11195 (Fig. 3). Reactive astrocytes and activated microglia were observed in the vicinity of Aβ plaques in the AD frontal cortex [89]. Microglial innate immune responses are highly versatile in AD, producing an array of proinflammatory cytokines and mediators in response to Aβ. These proinflammatory changes activates astrocytes, which in turn secretes cytokines such as interleukin-1 and TNF-alpha. Thus, a vicious neuroinflammatory cycle occurs that initiates and propels disease forward to widespread circuits that undergo dysfunction at a later stage.

Regions within the default-mode network (DMN) are highly overlapping with the spatial distribution of both Aβ and tau pathologies detected by using PET [90–94]. The degree of alterations to DMN connectivity has also been found relating to disease progression [92]. The spread of amyloid pathology from the medial temporal lobe has been found associated with glucose metabolism measured by ^18^F-FDG PET [95, 96].

This study presents the binding properties of different PET tracers for detecting different AD pathologies. We have to conclude that investigations in autopsy AD brain tissues represent the status at final stage of AD. This is in contrast to *in vivo* PET and MRI studies [3, 4], where the regional rate of deposit of different pathological markers including amyloid plaque load, tau, astrogliosis, and microglia activation, and neurodegeneration in form of hypoglucose metabolism and structural changes (atrophy) can be measured during the time course of the disease from preclinical stages, defined also by the ATN criteria [10]. In addition, there is a rapid development in the field of imaging biomarkers in AD for amyloid-beta, tauopathy [97, 98], neuroinflammation as well as synaptic density measurement [99]. Magnetic resonance imaging diffusion basis spectrum imaging has been used to detect indicators of neuroinflammation in AD, provide further insight into white matter microstructural integrity [100, 101].

There are several limitations of the study. Firstly, additional *TSPO* gene *rs6971* polymorphism data could be useful for categorizing ^3^H-FEMPA high/mixed/low-affinity binders. Secondly, with regards to astrocyte and microglia subtypes [82], immunohistochemical staining with multiple antibodies such as glutamate synthases for astrocyte and MHC-II for microglia, as well as morphology study for resting versus activating subtypes. Thirdly, difference between the binding properties measured in autoradiography and homogenate assays remains unclear. One explanation is that TSPO and MAO-B, which both locates in the mitochondria, exposes to tracers at different degrees depending on the method adopted [65]. Parallel regional characterization with tau tracers and additional tracers for neuroinflammation will provide with a more comprehensive picture of the pathology in AD brain [69, 70, 102]. Fourthly, regional binding studies with tau PET tracers would have most probably provided valuable data especially regarding binding properties in AD hippocampus and temporal cortices. Unfortunately, we did not have access to any ^3^H-labelled tau tracer when the studies were performed.

## CONCLUSIONS

Our results showed a different regional profile of Aβ plaque deposits versus astrogliosis and microgliosis; and supported the specific measurement by ^3^H-florbetaben, ^3^H-L-deprenyl, GFAP expression, and ^3^H-PK11195 binding in AD and control brains. A clear relationship was observed between Aβ plaque deposition and astrogliosis in AD hippocampus which was not observed for microgliosis. Developing of more specific tracers for disease-relevant gliosis will improve the development of new PET tracers for studies of neuroglia in AD and thereby provide a further understanding of early disease mechanisms important early detection and diagnosis and new treatment targets in AD.

## Supplementary Material

Supplementary Material
