# Supplementary material for: In vitro Characterization of the Regional Binding Distribution of Amyloid PET Tracer Florbetaben and the Glia Tracers Deprenyl and PK11195 in Autopsy Alzheimer’s Brain Tissue
Source: J Alzheimers Dis. 2021 Apr 20;80(4):1723–37. doi: 10.3233/JAD-201344 (PMC8150513; doi:10.3233/JAD-201344)
Supplement: Supplementary Material [file jad-80-jad201344-s001.pdf]

Supplemental Material

***In vitro* Characterization of the Regional Binding Distribution of Amyloid PET Tracer Florbetaben and the Glia Tracers Deprenyl and PK1195 in Autopsy Alzheimer's Brain Tissue**

Methods

Immunohistochemistry

Sections from the frontal cortex and hippocampus of four AD and four control cases were double stained with Aβ antibody 4G8 ($Aβ_{17-24}$, 1:200 Signet/Covance, Princeton, NJ, USA) in combination with anti-GFAP (1:500, Dako, Denmark) or with anti-ionized calcium-binding adaptor molecule 1 (Iba) (1:500, Wako, Osaka, Japan). Immuno-reactivity was visualized using AP and horseradish peroxidase (HRP) systems (Invitrogen, La Jolla, CA, USA) and evaluated at 4 x magnification using a light microscope (Leica, Wetzlar, Germany) with an attached ProgRes® video camera and ProgRes®Capture Pro2.8.8 image analysis system (Jenoptik, Jena, Germany). Quantification was performed using ImageJ software (NIH, Bethesda, MD, USA). Results were presented as immunopositive cells/ mm^2.

Results

Immunohistochemical analysis of Aβ plaques, reactive astrocytes, and microglia in AD and control brain

To understand the imaging tracer binding-histopathology relationship, double immunohistochemical staining using 4G8 (for $Aβ_{17-24}$) antibodies followed by anti-GFAP or anti-Iba1 antibodies were performed in brain slices from the frontal cortex and the hippocampus of four AD and four control cases. Supplementary Figure 3 shows representative staining of 4G8/GFAP and 4G8/Iba1 in the slices from the frontal cortex and the hippocampus of one AD case. Greater numbers of Aβ deposits, Aβ-associated GFAP+ astrocytes, and Aβ-associated Iba1+ microglia were found in the frontal cortex (p = 0.0286, p = 0.0286, p = 0.0286 respectively) and the hippocampus (p = 0.0286, p = 0.0286, p = 0.0286 respectively) of AD (n = 4) compared to control cases (n = 4) (Supplementary Figure 3e-g).

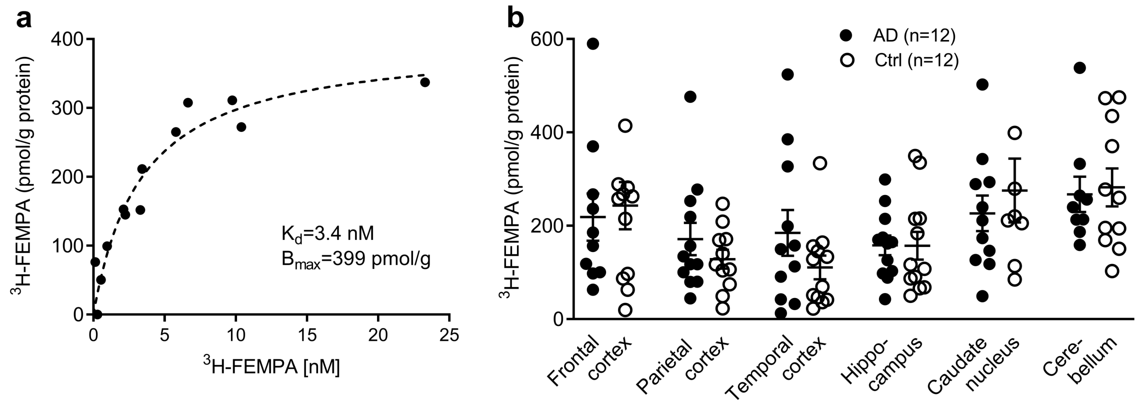

Supplementary Figure 1. a) Saturation binding curve for 3H-FEMPA in increasing concentration up to 25 nM. b) Regional binding of 3H-FEMPA in 6 different brain regions from 12 AD and 12 controls .

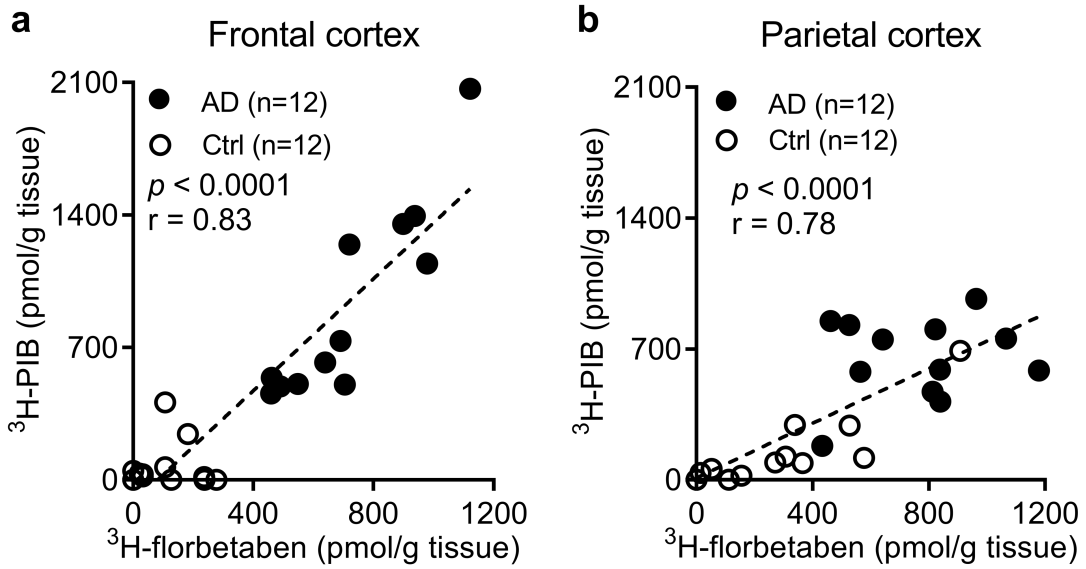

Supplementary Figure 2. a) Positive correlation between 3H-florbetaben and 3H-PIB binding in frontal cortex from 12 AD and 12 control brains. b) Positive correlation between 3H-florbetaben and 3H-PIB binding in parietal cortex from 12 AD and 12 control brains.

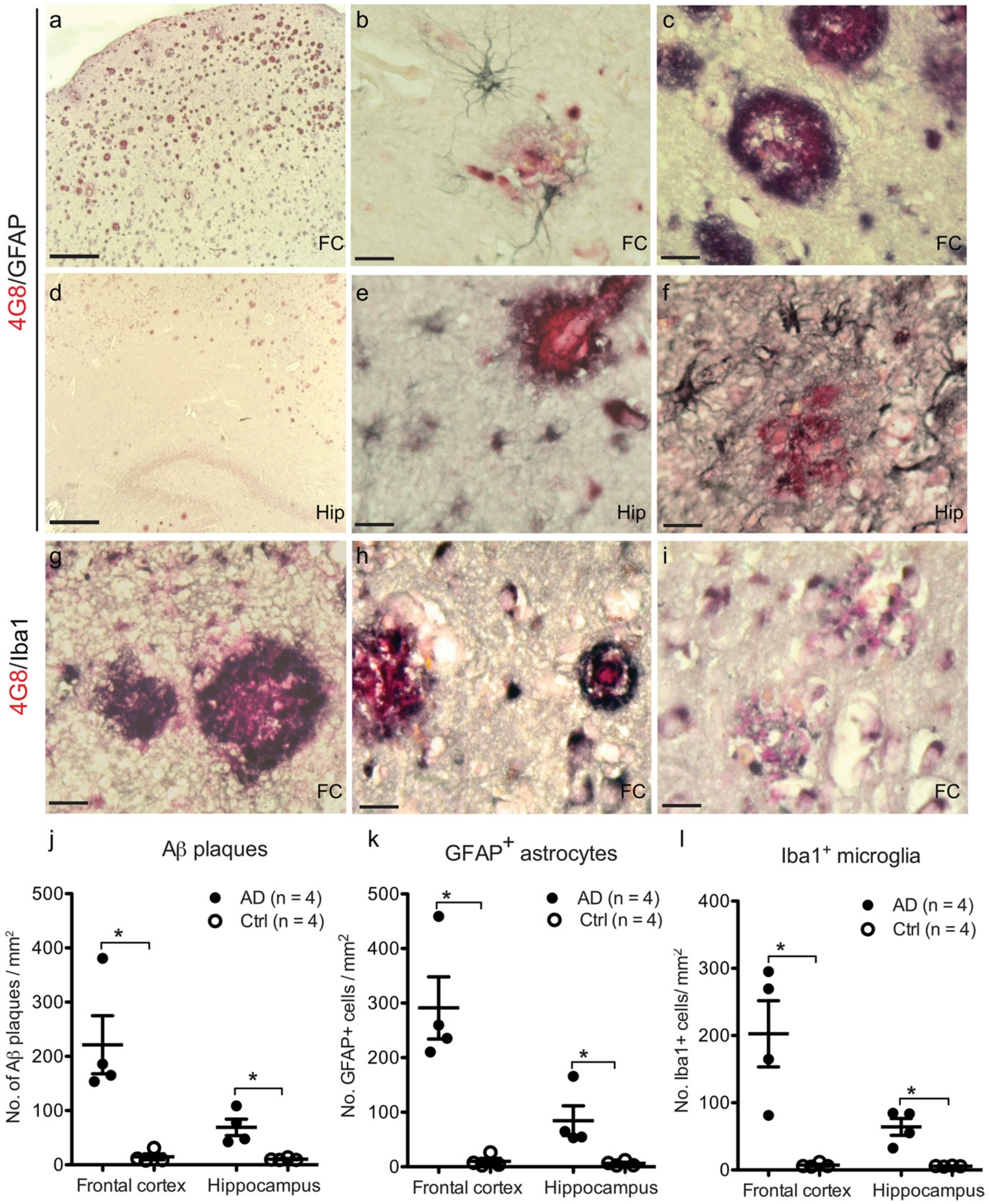

Supplementary Figure 3. Representative staining of 4G8/GFAP and 4G8/Iba1 in the slices from the frontal cortex and the hippocampus of one AD case. Greater numbers of Aβ deposits, Aβ-associated GFAP+ astrocytes and Aβ-associated Iba1+ microglia were found in the frontal cortex ($p = 0.0286$, $p = 0.0286$, $p = 0.0286$, respectively) and the hippocampus ($p = 0.0286$, $p = 0.0286$, $p = 0.0286$, respectively) of AD ($n = 4$) compared to control cases ($n = 4$) (e-g).
